# Evaluation of the chromogenic anti-factor IIa assay to assess dabigatran exposure in geriatric patients with atrial fibrillation in an outpatient setting

**DOI:** 10.1186/s12959-016-0084-2

**Published:** 2016-05-06

**Authors:** Luigi Brunetti, Betty Sanchez-Catanese, Leonid Kagan, Xia Wen, Min Liu, Brian Buckley, James P. Luyendyk, Lauren M. Aleksunes

**Affiliations:** Department of Pharmacy Practice and Administration, Ernest Mario School of Pharmacy, Rutgers, The State University of New Jersey, Piscataway, USA; Department of Medicine, Robert Wood Johnson University Hospital-Somerset, Somerville, USA; Department of Pharmaceutics, Ernest Mario School of Pharmacy, Rutgers, The State University of New Jersey, Piscataway, USA; Department of Pharmacology and Toxicology, Ernest Mario School of Pharmacy, Rutgers, The State University of New Jersey, Piscataway, USA; Chemical Analytical Core Laboratory, Environmental and Occupational Health Sciences Institute, Rutgers, The State University of New Jersey, Piscataway, USA; Pathology and Diagnostic Inv., Michigan State University, East Lansing, USA

**Keywords:** Dabigatran, Atrial fibrillation, Geriatric, HPLC-MS/MS, Chromogenic anti-factor IIa

## Abstract

**Background:**

Dabigatran etexilate may be underutilized in geriatric patients because of inadequate clinical experience in individuals with severe renal impairment and post-marketing reports of bleeding events. Assessing the degree of anticoagulation may improve the risk:benefit ratio for dabigatran. The aim of this prospective study was to identify whether therapeutic drug monitoring of dabigatran anticoagulant activity using a chromogenic anti-factor IIa assay is a viable option for therapy individualization.

**Methods:**

Plasma dabigatran concentration was assessed in nine patients with nonvalvular atrial fibrillation aged 75 years or older currently receiving dabigatran etexilate for prevention of stroke, using an anti-factor IIa chromogenic assay and HPLC-MS/MS. Trough concentrations were evaluated on two separate occasions to determine intrapatient variation.

**Results:**

Blood was collected at 13.1 ± 2.3 h (mean ± SD) post dose from patients prescribed dabigatran etexilate 150 mg twice daily (5/9 patients) or dabigatran etexilate 75 mg twice daily (4/9 patients). Results from the anti-factor IIa chromogenic assay correlated with dabigatran concentrations as assessed by HPLC-MS/MS (*r*^*2*^ = 0.81, *n* = 16). There was no correlation between dabigatran trough values taken at separate visits (*r*^*2*^ = 0.002, *n* = 7). Furthermore, there was no correlation found between the drug concentrations and patients’ renal function determined by both creatinine and cystatin-C based equations. None of the patients enrolled in the study were in the proposed on-therapy trough range during at least one visit.

**Conclusion:**

The chromogenic anti-factor IIa assay demonstrated similar performance in quantifying dabigatran plasma trough concentrations to HPLC-MS/MS. Single measurement of dabigatran concentration by either of two methods during routine visits may not be reliable in identifying patients at consistently low or high dabigatran concentrations.

**Electronic supplementary material:**

The online version of this article (doi:10.1186/s12959-016-0084-2) contains supplementary material, which is available to authorized users.

## Background

Dabigatran possesses many of the attributes of an ideal anticoagulant for stroke prevention in nonvalvular atrial fibrillation (NVAF) including predictable pharmacokinetics and lack of the requirement for routine monitoring [1–3]. While routine monitoring may be unnecessary, assessment of degree of anticoagulation may be important in populations at risk of altered pharmacokinetics [4, 5]. Since the FDA approval of dabigatran etexilate in 2010, several regulatory agencies have issued warnings regarding the risk of bleeding, analogous to other target specific oral anticoagulants and vitamin K antagonists. The majority of hemorrhagic events linked to dabigatran have been reported in geriatric patients with renal dysfunction [6–9]. Although the landmark Randomized Evaluation of Long-Term Anticoagulation Therapy (RE-LY) trial found dabigatran etexilate 150 mg twice daily to be superior to warfarin; it has been difficult to extrapolate the results to the geriatric population or to patients with severe renal impairment. A post-hoc analysis of the RE-LY trial revealed that patients ≥ 75 years of age had a greater incidence of gastrointestinal bleeding (but not intracranial) compared with patients on warfarin (1.85 %/year versus 1.25 %/year, respectively, *p* < 0.001) [10]. Furthermore, an increased risk of bleeding was identified in elderly patients irrespective of renal function [11]. Dabigatran etexilate is underutilized in geriatric patients because of insufficient clinical experience with dosing recommendations in severe renal impairment and post-marketing reports of bleeding complications [6–8, 12–18]. The mean age of RE-LY patients was 71.5 years old and the mean creatinine clearance (CrCl) was approximately 70 mL/min [19]. Patients with a CrCl < 30 mL/min were excluded from RE-LY. Moreover, the FDA approval of dabigatran etexilate dosing regimen for patients with severe renal dysfunction was supported by pharmacokinetic modeling based on data from middle-aged patients rather than actual clinical outcome [20–23]. The European Medicines Agency (EMA) considers dabigatran etexilate as contraindicated in patients with a CrCl < 30 mL/min and patients with a CrCl < 50 mL/min should receive 110 mg twice daily [24]. Collectively, these data suggest that the ability to gauge the degree of anticoagulation in the geriatric patient population may be beneficial.

There are a number of routine coagulation tests used in clinical practice; however, few are useful for quantitative assessment of dabigatran [5, 25]. The chromogenic anti-factor IIa assay has been successfully used for therapeutic drug monitoring of parenteral direct thrombin inhibitors and is insensitive to lupus anticoagulant or genetic coagulation deficiencies [26, 27]. Very little data have been published on the use of chromogenic anti-factor IIa assay and its correlation with HPLC-MS-MS measurement of dabigatran [28]. The aim of this prospective pilot study was to evaluate the utility of the chromogenic anti-factor IIa assay for monitoring dabigatran therapy and the intra- and interpatient variability of trough concentrations in elderly patients with atrial fibrillation.

## Methods

A prospective study of nine geriatric patients was performed to assess dabigatran plasma trough concentrations using HPLC-MS/MS and the chromogenic anti-factor IIa quantification methods on two separate visits to the clinic. Male and female patients ≥ 75 years of age with NVAF currently receiving dabigatran etexilate mesylate (dabigatran prodrug) for the prevention of stroke were eligible for inclusion. Patients with a creatinine clearance of less than 15 mL/min were excluded since data are extremely limited and the use of dabigatran etexilate is contraindicated in this population (based on the United States product labeling) [29]. Patients with hemorrhagic disorders or baseline platelet count of less than 100,000 per liter, on hemodialysis, or with moderate or severe liver impairment (Child Pugh Score of B or greater) or those on strong P-glycoprotein inhibitors and inducers (i.e., amiodarone, clarithromycin, dronederone, ketoconazole, quinidine, rifampin, verapamil, and St. John’s wort) were excluded. Dabigatran etexilate should be avoided with rifampin due to significant reduction in area under the curve (AUC) and maximum serum concentration (C_max_) (66 and 67 %, respectively) [29]. While not contraindicated with P-glycoprotein inhibitors, the use of dabigatran etexilate with these agents should be carefully monitored due to increased AUC and C_max_. Furthermore, in the setting of moderate-to-severe renal dysfunction and a P-glycoprotein inhibitor, dabigatran etexilate dose reductions should be considered [29]. The protocol was approved by the Rutgers University Institutional Review Board (Protocol # 13–503) and all patients signed an informed consent before participating in the study.

### Patient dosing

The morning of study initiation, consenting patients were instructed to hold the morning dabigatran etexilate dosage until a blood sample was obtained at the physician’s office. Once venous blood samples were drawn, the patient was instructed to take his/her dose. Patient demographics and concomitant medications were collected. The process was repeated on the patient’s next scheduled visit, a minimum of 1 month apart.

### Sample collection

Venous blood samples were taken just prior to the morning dose. Approximately 5 mL was collected in EDTA tubes for dabigatran plasma concentration measurement by HPLC-MS/MS. Another 5 mL was collected in 3.2 % tri-sodium citrate tubes (blood:citrate ratio 9:1) as recommended by the manufacturer for chromogenic assay. The samples were centrifuged at 2500 × g for 20 min and the plasma was kept on ice for a max of 1 h. Samples were kept frozen at –80 °C until assessment.

### Quantitation of dabigatran

Dabigatran concentration in plasma samples was directly measured using a validated HPLC-MS/MS technique (modified from Delavenne et al.) [30] and estimated using a chromogenic anti-factor IIa assay (Hyphen Biomed, Neuville-sur-Oise, France). Plasma samples or standards (100 μL) were mixed with 10 μL of an internal standard (^13^C_6_-dabigatran 1 μg/mL). Analytes were isolated from plasma using protein precipitation with 400 μL methanol/0.1 N HCl (90:10). After centrifugation, a 100 μL aliquot of supernatant was taken for the injection, and the injection volume was 20 μL. A Thermo LTQ mass spectrometer was interfaced to a Finnigan Surveyor Autosampler plus and Finnigan Surveyor MS Pump plus for separation and quantitation of dabigatran. Separation was completed using Betasil Phenyl/Hexyl column (3 μm, 100 × 4.6 mm, Thermo Scientific) and a gradient flow of water and methanol with 0.1 % formic acid. Electrospray ionization source was used to ionize the dabigatran before introduction into the mass spectrometer. Quantification was performed by addition of 472.2– > 324.2 and 472.2– > 306.1 and 472.2– > 289.1 m/z for dabigatran and 478.3– > 330.2 and 478.3– > 295.1 m/z for the internal standard. The calibration curves were linear over a concentration range of 4–1000 ng/mL.

### Chromogenic anti-IIa assay

Dabigatran activity was quantified using a BIOPHEN DTI kit (Aniara, West Chester, OH). Plasma samples, dabigatran calibrators or quality controls (50 μl) were mixed with 50 μl of thrombin chromogenic substrate at 37 °C for 1 min in a 96-well plate. The mixture was then incubated at 37 °C for 2 min after adding 50 μl of pre-heated purified human thrombin. Activity was measured spectrophotometrically at 450 nm (SpectraMax 5, Molecular Devices, Sunnyvale, CA) in the presence of 20 % of acetic acid and adjusted for sample blanks and extrapolated from a standard curve. Samples were run in duplicate. The limit of detection was 14.6 ng/mL and the dynamic range from 0 to 500 ng/mL.

### Assessment of renal function

Both serum creatinine and cystatin-C were measured in order to estimate renal function using the Cockcroft-Gault ([140 – age [years] × total body weight]/0.72 × sCr (mg/dL)) × 0.85 [if female]) and CKD-EPI (127.7 × Cystatin C^-1.17^ × age^-0.13^ × 0.91 [if female] × 1.06 [if African American]) equations, respectively [31, 32]. Of note, Cockcroft-Gault was the method used to estimate renal function in RE-LY, [19] the landmark trial leading to the approval of dabigatran etexilate for prevention of stroke and systemic embolism in patients with NVAF. Serum creatinine levels were measured using a kit based on the Jaffe reaction (Pointe Scientific, Canton, MI). Briefly, 190 μl of pre-heated working reagent including 5 volumes of alkaline buffer and 1 volume of picric acid (40 mM) were added to 10 μl of samples, creatinine standard or blank serum. The mixture was incubated at 37 °C for 1 min and the change in optical density was measured at 510 nm over 3 min.

Cystatin C levels were quantified using a Quantikine ELISA kit according to the manufacturer’s recommendations (R&D Systems, Minneapolis, MN). Samples or cystatin C standards (50 μl) were added to a 96-well plate coated with an antibody specific for human cystatin C and incubated at 2–8 °C for 3 h. After washing, cystatin C conjugate was then added to compete for binding with the antibody. Following incubation, washing and addition of substrate solutions (stabilized hydrogen peroxide and tetramethylbenzidine), the stop solution (2 N sulfuric acid) was added and the optical density was measured at 450 nm and 570 nm. Concentrations of cystatin C were extrapolated from the standard curve. Samples were run in duplicate. Renal function was assessed at each visit.

### Data analysis

All data were analyzed using descriptive statistics. Categorical data were reported as proportions and continuous data as the mean or median as appropriate. Pearson correlation coefficients were calculated for the relationship between HPLC-MS/MS and chromogenic assay dabigatran trough levels and estimates of renal function. Bland-Altman analysis and linear regression were performed to assess the strength of agreement and proportionality bias between HPLC-MS/MS and chromogenic anti-IIa measures of dabigatran levels. Correlation of dabigatran trough levels between visits was also evaluated. Trough levels were also compared to proposed dabigatran on target range (30 ng/mL – 130 ng/mL) [33]. Analysis was performed using SAS 9.2 (SAS Institute, Cary, NC) or SPSS version 21 (IBM Corporation, Armonk, NY).

## Results

**Fig. 1:**
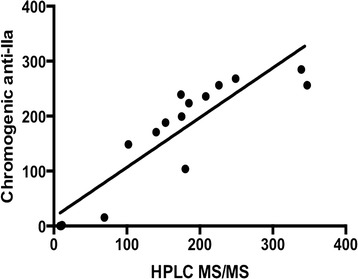
The relationship between plasma dabigatran concentrations determined by chromogenic anti-IIa assay and HPLC-MS-MS. Solid line – linear regression y = 0.9053x + 16.11, *r* ^*2*^ = 0.81

**Fig. 2:**
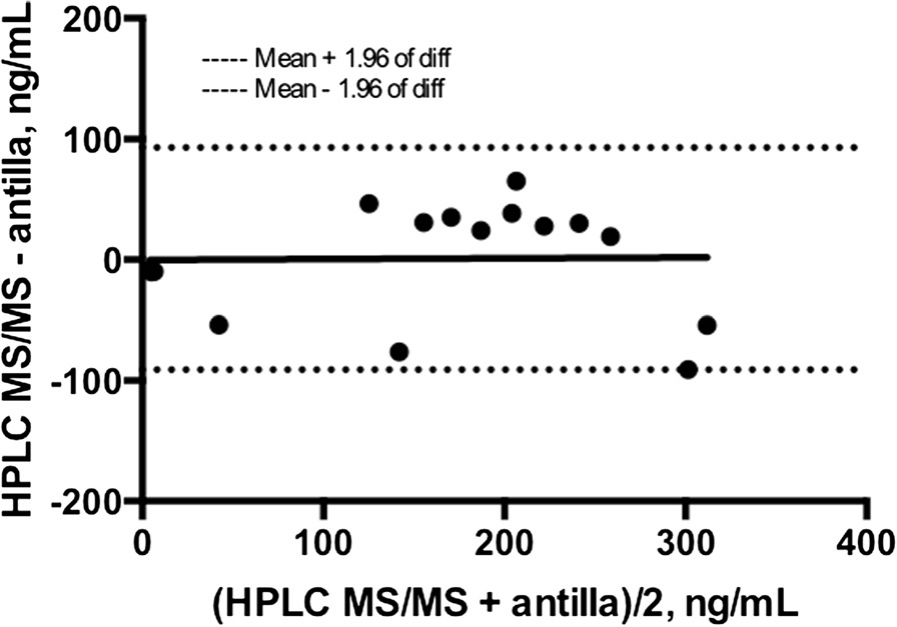
Bland-Altman plot is shown for dabigatran levels by HPLC MS/MS and chromogenic anti-factor IIa (diff, difference; *n* = 16)

**Fig. 3:**
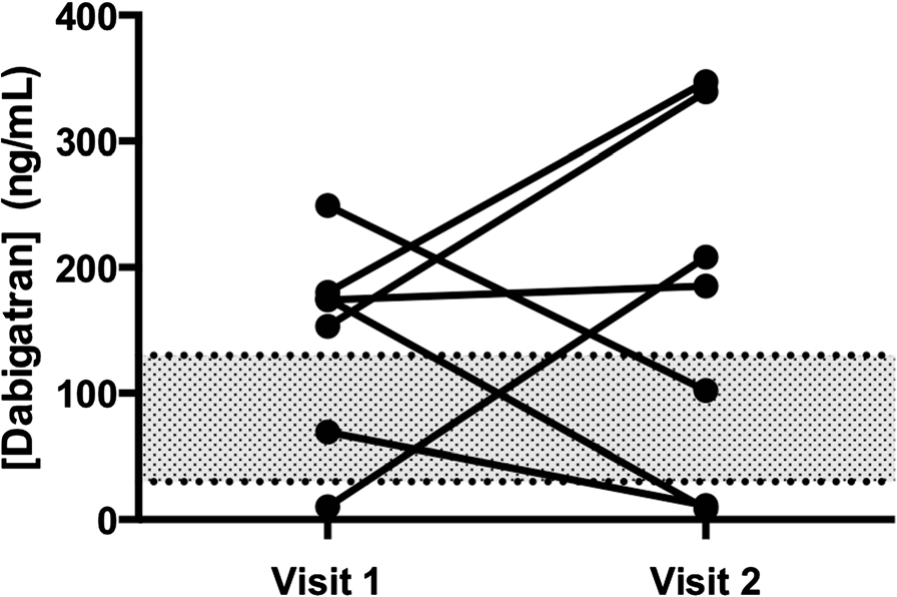
Individual dabigatran plasma trough concentration measured on two separate occasions using HPLC MS-MS (*n* = 7). High inter- and intrapatient variability was observed. Conceptually, due to this variability, the clinical application of therapeutic drug monitoring is challenging. Shaded area represents the on-therapy range (30 ng/mL to 130 ng/mL)

Nine patients were enrolled, seven patients returned for a second visit. All patients were on dabigatran etexilate therapy for a minimum of one month before initiation of the study. Patient characteristics are summarized in Table 1. Blood was collected at 13.1 ± 2.3 h (mean ± SD) post dose from patients receiving dabigatran etexilate 150 mg twice daily (5/9 patients) or dabigatran etexilate 75 mg twice daily (4/9 patients). Results from the anti-IIa chromogenic assay correlated with dabigatran concentrations as assessed by HPLC-MS/MS (*r*^*2*^ = 0.81, *n* = 15; Fig. 1). In addition, the Spearman’s rho yielded similar results (rho = 0.91). The Bland-Altman plot shows a very high limit of agreement defined by the mean ± 1.96*SD (Fig. 2). The mean bias present was 0.86 and the limits of agreement were 93.0 and – 91.0. The linear regression of the Bland-Altman plot did not suggest any significant proportionality bias (equation; Y = 0.006545*X – 0.1945; *p* = 0.9583). High intrapatient variability in dabigatran trough plasma concentrations was observed (*r*^2^ = 0.002, *p* = ns; *n* = 7; Fig. 3). All the patients enrolled in the study were not within the proposed on-therapy range [33] during at least one study visit. Seven patients had a dabigatran level exceeding 130 ng/mL and three patients had a level of less than 30 ng/mL during at least one of the recorded visits. Baseline creatinine based (Cockcroft-Gault) and cystatin-C based estimates (CKD-EPI) of renal function had no-to-poor correlation with plasma dabigatran concentrations (*r* = 0.07 and – 0.26, *p* = ns for both; respectively).

**Table 1:** Patient demographic and dabigatran dosing characteristics

Characteristic	Value
Mean Age ± SD (years)	81.3 ± 4.5
Female (%)	44.5
Mean time after last dabigatran dose ± SD (hours)	13.1 ± 2.3
Mean weight ± SD (kg)	83.0 ± 21.1
Body mass index ± SD (kg/m^2^)	28.9 ± 4.7
Baseline Renal Clearance ± SD (mL/min)	
Cockcroft-Gault	68.4 ± 28.4
CKD-EPI	40.9 ± 12.3
Dabigatran dosage, n (%)	
75 mg twice daily	4 (44.4)
150 mg twice daily	5 (55.6)
Cormorbidities (n, %)	
Chronic obstructive pulmonary disease	3 (33.3)
Diabetes Mellitus	4 (44.4)
Heart Failure	3 (33.3)
Malignancy	2 (22.2)
Thyroid Disease	4 (44.4)
Coronary Artery Disease	2 (22.2)
Mean HPLC-MS/MS dabigatran level ± SD (ng/mL)^a^	161.1 ± 104.1
Mean chromogenic anti-IIa dabigatran level ± SD (ng/mL)^a^	161.9 ± 104.8

^a^Pooled data from all office visits

## Discussion

There is a widely held view that the target specific oral anticoagulants, including dabigatran etexilate, have a predictable response and do not require monitoring; however, data suggest significant interpatient variability in pharmacokinetics [34, 35]. In addition, the landmark RE-LY trial suggests low trough concentrations (rapid decrease in the probability of stroke from a concentration of zero through approximately 70 ng/mL) [36, 37] were associated with reduced efficacy and high concentrations were associated with an increased risk of bleeding [4]. Chan and colleagues measured the Hemoclot® assay at baseline and every 2 months for up to 4 visits in 100 patients (mean age 69.9 years) with atrial fibrillation [35]. They reported a large intrapatient variability in Hemoclot® levels (geometric coefficient of variation 32 – 40 %). The authors concluded that a single Hemoclot® measurement is not reliable in identifying patients with consistently high or low dabigatran exposure. Some concerns have been raised regarding the large variation in trough dabigatran levels seen between visits in the Chan and colleagues study [38]. These concerns included timing of trough sample, stability of plasma stored at -80 °C, performance of the analysis on the same run, and lack of outcome data. Arguably, the most important concern is the lack of stringent timing of trough levels. While measuring trough levels at 12 ± 1 h is ideal, when relying on patient reported drug administration in clinical practice this criteria is difficult to enforce. Patient reported adherence is inherently a limitation and may result in measurement bias. Similar to the Chan and colleagues study, our pilot study found that in geriatric patients there was large intrapatient variability in dabigatran exposure as measured by chromogenic anti-IIa assay and HPLC MS/MS. Specifics on last dose intake in the current study may be found in Additional file 1.

Vulnerable populations such as the elderly and patients with renal impairment have the potential to exhibit exaggerated responses to dabigatran [11, 39, 40]. Dabigatran etexilate, a prodrug, completes its bioconversion in the liver and approximately 20 % is conjugated with glucuronic acid and excreted via the biliary system [2, 41]. Dabigatran etexilate requires conversion via esterase hydrolysis to the active form (dabigatran) [2]. Genetic factors, such as polymorphisms in carboxylesterase 1, may also be responsible for interpatient variability [42]. There may also be variability in drug exposure secondary to inhibition or induction of the efflux transporter P-glycoprotein, as dabigatran etexilate is substrate of this transport protein [29, 43]. While these factors explain the interpatient variability that may be present, they do not account for the intrapatient variability observed between clinic visits in this study.

No therapeutic range has been established for dabigatran; however, a target plasma dabigatran trough concentration of 30 – 130 ng/mL has been suggested by Chin and colleagues [33]. Some limitations to using this range include derivation from pharmacokinetic simulations and lack of prospective studies confirming that the range predicts clinical outcomes. However, with the lack of definitive data, this range provides a good starting point and there are data from landmark trials confirming dabigatran levels may be predictive of thrombosis and bleeding [4]. For example, patients in the RE-LY trial with any major bleeding had a higher dabigatran trough concentration (113 ng/mL) compared to patients without a bleeding event (72.8 ng/mL) [4]. Furthermore, age was the most important covariate. Collectively, these data may be used to construct a dabigatran concentration-to-assay result curve to predict drug exposure and predict risk of bleeding [44]. In our analysis, we found that all patients were not in the on-therapy range on at least one of the two visits. Furthermore, 4 out of 9 patients had dabigatran trough levels exceeding 200 ng/mL during at least one visit and trough levels above 200 ng/mL are associated with an increased risk of bleeding [45]. These results are concerning and suggest geriatric patients may be at an unecessary risk of treatment failure and/or bleeding.

Estimating renal function in the elderly is challenging and many of the currently available methods are inaccurate [46]. Unlike creatinine, cystatin C levels are unaffected by age, muscle mass, gender, and race [32]. We were not able to appreciate any significant correlation with either creatinine or cystatin C based estimates of renal function with dabigatran trough concentrations. Based on this finding, additional research is warranted to identify which estimate renal function leads to the selection of the most appropriate dose or if age alone is sufficient to suggest a dosage reduction [47, 48]. Current FDA and EMA recommendations for dosing dabigatran etexilate in renal disease advocate using the Cockcroft-Gault equation to estimate renal function and clinicians should not deviate from this strategy [24, 29]. Hellden and colleagues investigated the impact of using the Modified Diet in Renal Disease 4 (MDRD4 equation to estimate glomerular filtration rate and subsequent dose adjustment in the elderly population (defined as age > 65 years) [49]. Their findings suggest that the MDRD4 would result in higher recommended doses of dabigatran etexilate to elderly patients versus Cockcroft-Gault, particularly in women. The increased dose may increase the risk of toxicity, hence these findings suggest continued use of Cockcroft-Gault to estimate renal function for dabigatran etexilate dosing.

These data support further evaluation of strategies to individualize treatment. The literature on coagulation monitoring to guide dabigatran therapy is evolving with several studies and comprehensive reviews now published [4, 5, 34, 44, 50–55]. Evidence supports that dabigatran levels are correlated to bleeding risk and efficacy [4]. Furthermore, in an sub-analysis of the RE-LY trial, a plasma concentration at trough between 90 and 140 ng/mL provided the best benefit/risk ratio in patients with NVAF,[56] although other authors have suggested other on-target ranges [33, 57]. Tailoring dabigatran etexilate dose according to patient risk (i.e., age, renal function) is essential to balance the benefit:risk of thrombosis and bleeding [58]. Adding the ability to assess degree of anticoagulation has the potential to further improve the benefit:risk ratio of dabigatran and warrants consideration especially in special populations such as the geriatric population [59, 60].

This study provides important information obtained from ‘real world’ use of dabigatran etexilate in geriatric patients. Chromogenic anti-IIa assay correlates with HPLC MS/MS measured dabigatran concentrations and may be useful for quantitative measurement; however, the intrapatient variability of dabigatran concentrations may make clinical application challenging. The frequency of patients outside a proposed therapeutic window suggests there may be opportunity for improvement of dosing strategy to further enhance the risk versus benefit ratio of dabigatran. Glucuronidation is the major metabolic pathway of dabigatran. The major metabolite of dabigatran, 1-O-acylglucuronide, and its isomers result in equipotent prolongation of the activated partial thromboplastin time [41, 61]. Acylglucuronides accounted for 2.0 % of the dose in plasma at 2 h and 4.3 % at 4 h post administration of intravenous dabigatran [41]. The acylglucuronide metabolites may contribute to the overall clinical effect of dabigatran and can explain some of the difference between HPLC-MS/MS detection of dabigatran and the chromogenic measurement of anti-IIa activity if there is interpatient variability in glucuronidation. Of note, previous studies suggest that age does not significantly influence glucuronidation [62, 63].

Certain limitations of our study should be acknowledged. Although the chomogenic anti-factor II assay may be performed manually or using an automated coagulometer as indicated in the assay specifications, manual methods may be a potential source of measurement bias. The timing of trough levels was often not within 1 h of the next scheduled dose due to patient availability, as suggeted to be optimal for pharmacokinetic studies [50]. Our data reflects a practical scenario that resembles the ‘real world’ clinical setting. Furthermore, data support that sampling within 6 h of the next scheduled dose will still provide a value within the 80 % confidence interval for the true trough value as was discussed by Chan and colleagues [22]. When planning to measure dabigatran levels it is paramount to educate the patient on the importance of accurately documenting the last intake of medication. In addition, scheduling patient visits according to their usual drug administration schedule may enhance the accuracy of trough levels. Another strategy involves collaboration of clinicians with laboratories or anticoagulation clinics. Patients can be instructed to hold their dabigatran etexilate dose until their office visit where administration can be directly observed. Following directly observed administration of dabigatran etexilate, the office staff can schedule an appointment for the patient to present to the laboratory or clinic for their blood to be drawn.

This study found no correlation between dabigatran trough levels taken at two different patient visits; however, the limited sample size requires future studies to confirm this finding. Ultimately, a large controlled study is necessary to confirm if a monitoring strategy will improve dosage selection and dabigatran treatment outcomes.

## Conclusion

Chromogenic anti-factor IIa assay demonstrated similar performance in quantifying dabigatran plasma trough concentrations to HPLC-MS/MS. All geriatric patients were not within the on-therapy trough range during at least one visit. Routine adjustment of dosages based on a single measurement of trough concentration may not be appropriate due to significant intrapatient variation. Given the large proportion of patients falling outside the on-therapy range and the high variability observed in this pilot study, larger clinical studies can be recommended to determine the clinical utility of concentration monitoring in the outpatient setting.

## Additional file

Additional file 1: Individual patient characteristics. (DOCX 61 kb)

## References

[1] Hankey GJ (2009). At last, a RE-LYable alternative to warfarin for atrial fibrillation. Int J Stroke.

[2] Hankey GJ, Eikelboom JW (2011). Dabigatran etexilate: a new oral thrombin inhibitor. Circulation.

[3] Moore TJ, Cohen MR, Mattison DR. Dabigatran, bleeding, and the regulators. BMJ. 2014;349:g4517. http://dx.doi.org/10.1136/bmj.g4517. 10.1136/bmj.g4517 25056265

[4] Reilly PA, Lehr T, Haertter S, Connolly SJ, Yusuf S, Eikelboom JW (2014). The effect of dabigatran plasma concentrations and patient characteristics on the frequency of ischemic stroke and major bleeding in atrial fibrillation patients: The RE-LY Trial (Randomized Evaluation of Long-Term Anticoagulation Therapy). J Am Coll Cardiol.

[5] Brunetti L, Bandali F (2013). Dabigatran: is there a role for coagulation assays in guiding therapy?. Ann Pharmacother.

[6] Harper P, Young L, Merriman E (2012). Bleeding risk with dabigatran in the frail elderly. N Engl J Med.

[7] Legrand M, Mateo J, Aribaud A, Ginisty S, Eftekhari P, Huy PT (2011). The use of dabigatran in elderly patients. Arch Intern Med.

[8] Safouris A, Triantafyllou N, Parissis J, Tsivgoulis. The case for dosing dabigatran: how tailoring dose to patient renal function, weight, and age could improve the benefit-risk ratio. Ther Adv Neurol Disord. 2015;8(6):245-54. doi:10.1177/1756285615601360. 10.1177/1756285615601360 PMC4643866 26600870

[9] Cotton BA, McCarthy JJ, Holcomb JB (2011). Acutely injured patients on dabigatran. N Engl J Med.

[10] Eikelboom JW, Connolly SJ, Hart RG, Wallentin L, Reilly P, Oldgren J (2013). Balancing the benefits and risks of 2 doses of dabigatran compared with warfarin in atrial fibrillation. J Am Coll Cardiol.

[11] Eikelboom JW, Wallentin L, Connolly SJ, Ezekowitz M, Healey JS, Oldgren J (2011). Risk of bleeding with 2 doses of dabigatran compared with warfarin in older and younger patients with atrial fibrillation: an analysis of the randomized evaluation of long-term anticoagulant therapy (RE-LY) trial. Circulation.

[12] Radecki RP (2012). Dabigatran: uncharted waters and potential harms. Ann Intern Med.

[13] Anonymous. Bleeding with dabigatran (Pradaxa). Med Lett Drugs Ther. 2011;53(1379-1380):98. 22173427

[14] Barton CA, McMillian WD, Sadi Raza S, Keller RE (2012). Hemopericardium in a patient treated with dabigatran etexilate. Pharmacotherapy.

[15] Bene J, Said W, Rannou M, Deheul S, Coupe P, Gautier S (2012). Rectal bleeding and hemostatic disorders induced by dabigatran etexilate in 2 elderly patients. Ann Pharmacother.

[16] Cano EL, Miyares MA (2012). Clinical challenges in a patient with dabigatran-induced fatal hemorrhage. Am J Geriatr Pharmacother.

[17] Kernan L, Ito S, Shirazi F, Boesen K (2012). Fatal gastrointestinal hemorrhage after a single dose of dabigatran. Clin Toxicol (Phila).

[18] Lillo-Le Louet A, Wolf M, Soufir L, Galbois A, Dumenil AS, Offenstadt G (2012). Life-threatening bleeding in four patients with an unusual excessive response to dabigatran: implications for emergency surgery and resuscitation. Thromb Haemost.

[19] Connolly SJ, Ezekowitz MD, Yusuf S, Eikelboom J, Oldgren J, Parekh A (2009). Dabigatran versus warfarin in patients with atrial fibrillation. N Engl J Med.

[20] Hariharan S, Madabushi R (2012). Clinical pharmacology basis of deriving dosing recommendations for dabigatran in patients with severe renal impairment. J Clin Pharmacol.

[21] Lehr T, Haertter S, Liesenfeld KH, Staab A, Clemens A, Reilly PA (2011). Dabigatran etexilate in atrial fibrillation patients with severe renal impairment: dose identification using pharmacokinetic modeling and simulation. J Clin Pharmacol.

[22] Liesenfeld KH, Lehr T, Dansirikul C, Reilly PA, Connolly SJ, Ezekowitz MD (2011). Population pharmacokinetic analysis of the oral thrombin inhibitor dabigatran etexilate in patients with non-valvular atrial fibrillation from the RE-LY trial. J Thromb Haemost.

[23] Kowey PR, Naccarelli GV (2012). The food and drug administration decision not to approve the 110 mg dose of dabigatran: give us a way out. Am J Med.

[24] Pradaxa® European Medicines Agency. http://www.ema.europa.eu/docs/en_GB/document_library/EPAR_-_Product_Information/human/000829/WC500041059.pdf.

[25] van Ryn J, Stangier J, Haertter S, Liesenfeld KH, Wienen W, Feuring M (2010). Dabigatran etexilate--a novel, reversible, oral direct thrombin inhibitor: interpretation of coagulation assays and reversal of anticoagulant activity. Thromb Haemost.

[26] Salemi A, Agrawal YP, Fontes MA (2011). An assay to monitor bivalirudin levels on cardiopulmonary bypass. Ann Thorac Surg.

[27] Salmela B, Joutsi-Korhonen L, Saarela E, Lassila R (2010). Comparison of monitoring methods for lepirudin: impact of warfarin and lupus anticoagulant. Thromb Res.

[28] Adcock DM, Gosselin R, Kitchen S, Dwyre DM (2013). The effect of dabigatran on select specialty coagulation assays. Am J Clin Pathol.

[29] Boehringer Ingelheim. Pradaxa (dabigatran etexilate mesylate) prescribing information. http://docs.boehringer-ingelheim.com/Prescribing%20Information/PIs/Pradaxa/Pradaxa.pdf. (Accessed 2016 Apr 26).

[30] Delavenne X, Moracchini J, Laporte S, Mismetti P, Basset T (2012). UPLC MS/MS assay for routine quantification of dabigatran - a direct thrombin inhibitor - in human plasma. J Pharm Biomed Anal.

[31] Dowling TC, Wang ES, Ferrucci L, Sorkin JD (2013). Glomerular filtration rate equations overestimate creatinine clearance in older individuals enrolled in the Baltimore longitudinal study on aging: impact on renal drug dosing. Pharmacotherapy.

[32] Hojs R, Bevc S, Ekart R, Gorenjak M, Puklavec L (2008). Serum cystatin C-based equation compared to serum creatinine-based equations for estimation of glomerular filtration rate in patients with chronic kidney disease. Clin Nephrol.

[33] Chin PK, Wright DF, Patterson DM, Doogue MP, Begg EJ (2014). A proposal for dose-adjustment of dabigatran etexilate in atrial fibrillation guided by thrombin time. Br J Clin Pharmacol.

[34] Freyburger G, Macouillard G, Labrouche S, Sztark F (2011). Coagulation parameters in patients receiving dabigatran etexilate or rivaroxaban: two observational studies in patients undergoing total hip or total knee replacement. Thromb Res.

[35] Chan NC, Coppens M, Hirsh J, Ginsberg JS, Weitz JI, Vanassche T (2015). Real-world variability in dabigatran levels in patients with atrial fibrillation. J Thromb Haemost.

[36] US Food and Drug Administration, Center for Drug Evaluation and Research. Dabigatran etexilate; deputy office director decisional memo application 22-512. October 19, 2010. http://www.Accessdata.Fda.Gov/drugsatfda_docs/nda/2010/022512orig1s000sumr.pdf Accessed 30 Oct 2013).

[37] US Food and Drug Administration, Center for Drug Evaluation and Research. Dabigatran etexilate. Advisory committee briefing document, August 27, 2010. http://www.Fda.Gov/downloads/advisorycommittees/committeesmeetingmaterials/drugs/cardiovascularandrenaldrugsadvisorycommittee/ucm226009.pdf Accessed 30 Oct 2013.

[38] Douxfils J, Chatelain B, Dogne JM, Mullier F (2015). Real-world variability in dabigatran levels in patients with atrial fibrillation: comment. J Thromb Haemost.

[39] Stangier J, Stahle H, Rathgen K, Fuhr R (2008). Pharmacokinetics and pharmacodynamics of the direct oral thrombin inhibitor dabigatran in healthy elderly subjects. Clin Pharmacokinet.

[40] Stangier J, Rathgen K, Stahle H, Mazur D (2010). Influence of renal impairment on the pharmacokinetics and pharmacodynamics of oral dabigatran etexilate: an open-label, parallel-group, single-centre study. Clin Pharmacokinet.

[41] Blech S, Ebner T, Ludwig-Schwellinger E, Stangier J, Roth W (2008). The metabolism and disposition of the oral direct thrombin inhibitor, dabigatran, in humans. Drug Metab Dispos.

[42] Pare G, Eriksson N, Lehr T, Connolly S, Eikelboom J, Ezekowitz MD (2013). Genetic determinants of dabigatran plasma levels and their relation to bleeding. Circulation.

[43] Nutescu E, Chuatrisorn I, Hellenbart E (2011). Drug and dietary interactions of warfarin and novel oral anticoagulants: an update. J Thromb Thrombolysis.

[44] Avecilla ST, Ferrell C, Chandler WL, Reyes M (2012). Plasma-diluted thrombin time to measure dabigatran concentrations during dabigatran etexilate therapy. Am J Clin Pathol.

[45] Huisman MV, Lip GY, Diener HC, Brueckmann M, van Ryn J, Clemens A (2012). Dabigatran etexilate for stroke prevention in patients with atrial fibrillation: resolving uncertainties in routine practice. Thromb Haemost.

[46] Spruill WJ, Wade WE, Cobb HH (2008). Comparison of estimated glomerular filtration rate with estimated creatinine clearance in the dosing of drugs requiring adjustments in elderly patients with declining renal function. Am J Geriatr Pharmacother.

[47] Cockcroft DW, Gault MH (1976). Prediction of creatinine clearance from serum creatinine. Nephron.

[48] Chin P, Vella-Brincat J, Walker S, Barclay M, Begg E (2013). Dosing of dabigatran etexilate in relation to renal function and drug interactions at a tertiary hospital. Intern Med J.

[49] Hellden A, Odar-Cederlof I, Nilsson G, Sjoviker S, Soderstrom A, Euler M (2013). Renal function estimations and dose recommendations for dabigatran, gabapentin and valaciclovir: a data simulation study focused on the elderly. BMJ Open.

[50] Douxfils J, Lessire S, Dincq AS, Hjemdahl P, Ronquist-Nii Y, Pohanka A (2015). Estimation of dabigatran plasma concentrations in the perioperative setting. An ex vivo study using dedicated coagulation assays. Thromb Haemost.

[51] He S, Wallen H, Bark N, Blomback M (2013). In vitro studies using a global hemostasis assay to examine the anticoagulation effects in plasma by the direct thrombin inhibitors: dabigatran and argatroban. J Thromb Thrombolysis.

[52] Eikelboom JW, Weitz JI (2013). Dabigatran monitoring made simple?. Thromb Haemost.

[53] Douxfils J, Mullier F, Robert S, Chatelain C, Chatelain B, Dogne JM (2012). Impact of dabigatran on a large panel of routine or specific coagulation assays. Laboratory recommendations for monitoring of dabigatran etexilate. Thromb Haemost.

[54] Samama MM, Guinet C (2011). Laboratory assessment of new anticoagulants. Clin Chem Lab Med.

[55] Stangier J, Feuring M (2012). Using the HEMOCLOT direct thrombin inhibitor assay to determine plasma concentrations of dabigatran. Blood Coagul Fibrinolysis.

[56] Boehringer Ingelheim. An idea for a mid to long term strategy for Pradaxa, showing EMA range comparisons. 2014. Available from: http://journals.bmj.com/site/bmj/dabigatran/compared_ema.pdf [Cited 15 March 2016].

[57] Chin PK, Vella-Brincat JW, Barclay ML, Begg EJ (2012). Perspective on dabigatran etexilate dosing - why not follow standard pharmacological principles?. Br J Clin Pharmacol.

[58] Rosencher N, Albaladejo P (2012). A new approach with anticoagulant development: tailoring anticoagulant therapy with dabigatran etexilate according to patient risk. Expert Opin Pharmacother.

[59] Cohen D. Dabigatran: how the drug company withheld important analyses. BMJ. 2014;349:g4670. doi:http://dx.doi.org/10.1136/bmj.g4670. 10.1136/bmj.g4670 25055829

[60] Douxfils J, Mullier F, Dogne JM (2015). Dose tailoring of dabigatran etexilate: obvious or excessive?. Expert Opin Drug Saf.

[61] Ebner T, Wagner K, Wienen W (2010). Dabigatran acylglucuronide, the major human metabolite of dabigatran: in vitro formation, stability, and pharmacological activity. Drug Metab Dispos.

[62] Herd B, Wynne H, Wright P, James O, Woodhouse K (1991). The effect of age on glucuronidation and sulphation of paracetamol by human liver fractions. Br J Clin Pharmacol.

[63] Court MH (2010). Interindividual variability in hepatic drug glucuronidation: studies into the role of age, sex, enzyme inducers, and genetic polymorphism using the human liver bank as a model system. Drug Metab Rev.

