# Supplementary material for: Evaluation of the chromogenic anti-factor IIa assay to assess dabigatran exposure in geriatric patients with atrial fibrillation in an outpatient setting
Source: Thromb J. 2016 May 6;14:10. doi: 10.1186/s12959-016-0084-2 (PMC4858862; doi:10.1186/s12959-016-0084-2)
Supplement: Additional file 1: — Individual patient characteristics. (DOCX 61 kb) [file 12959_2016_84_MOESM1_ESM.docx]

| **Appendix 1: Individual patient characteristics** | | | | | | | | | | | | | |
| --- | --- | --- | --- | --- | --- | --- | --- | --- | --- | --- | --- | --- | --- |
| **Patient Characteristics** | | | | **Visit 1** | | | | | **Visit 2** | | | | |
| Age | Sex | Dose*  (mg) | BMI (kg/m^2^) | Last  dose (h) | HPLC-MS/MS level (ng/mL) | Chromogenic anti-factor IIa level (ng/mL) | CrCl (CG, ml/min) | CrCl (CKD-EPI, ml/min) | Last  dose (h) | HPLC-MS/MS level (ng/mL) | Chromogenic anti-factor IIa level (ng/mL) | CrCl (CG, ml/min) | CrCl (CKD-EPI, ml/min) |
| 77 | M | 75 | 35.1 | 15.5 | 175.0 | 199.0 | 121.0 | 37.4 | 14.5 | 8.5 | 0.0 | 83.1 | 37.0 |
| 76 | M | 150 | 28.9 | 15 | 249.0 | 268.1 | 63.8 | 49.3 | 15.0 | 102.0 | 148.5 | 66.4 | 46.2 |
| 82 | M | 150 | 26.7 | 13 | 153.0 | 188.1 | 74.5 | 58.2 | 11.0 | 339.0 | 284.9 | 62.8 | 52.1 |
| 87 | F | 75 | 22.5 | 11 | 10.2 | 0.0 | 32.5 | 29.6 | 14.0 | 208.0 | 235.9 | 21.8 | 27.6 |
| 75 | M | 150 | 37.6 | 7.5 | 226.0 | 256.3 | 102.8 | 32.4 | NA | NA | NA | NA | NA |
| 81 | F | 75 | 28.3 | 11.5 | 180.0 | 103.9 | 44.8 | 26.3 | 14.5 | 347.0 | 256.0 | 44.5 | 28.2 |
| 83 | M | 150 | 27.6 | 10.5 | 174.0 | 239.0 | 71.0 | 43.2 | 11.5 | 185.0 | 223.4 | 52.1 | 38.6 |
| 87 | F | 75 | 28.2 | 13 | 140.0 | 170.8 | 59.2 | 32.7 | NA | NA | NA | NA | NA |
| 84 | F | 150 | 24.9 | 15 | 69.2 | 15.5 | 46.3 | 59.1 | 15.0 | 11.3 | 1.6 | 48.3 | 72.2 |
| BMI=body mass index; CG=Cockcroft-Gault; CrCl=creatinine clearance; HPLC MS/MS=high performance liquid chromatography mass spectroscopy  *Note=dosed every 12 hours | | | | | | | | | | | | | |
